# Zinc oxide nanoparticles reduce biofilm formation, synergize antibiotics action and attenuate *Staphylococcus aureus* virulence in host; an important message to clinicians

**DOI:** 10.1186/s12866-022-02658-z

**Published:** 2022-10-11

**Authors:** Aliaa Abdelghafar, Nehal Yousef, Momen Askoura

**Affiliations:** Department of Microbiology and Immunology, Faculty of Pharmacy, Zagazig University, Zagazig, 44519 Egypt

**Keywords:** *Staphylococcus aureus*, Zinc oxide nanoparticles (ZnO-NPs), Antibiofilm, Pathogenesis

## Abstract

**Background:**

Biofilm-related infections are difficult to be treated because of higher resistance to antimicrobial agents. Current study aims to characterize the influence of zinc oxide nanoparticles (ZnO-NPs) on both *S. aureus* susceptibility to antibiotics and pathogenesis.

**Methods:**

The influence of ZnO-NPs on biofilm formation by *S. aureus* was characterized by the crystal violet and tube assay. The synergistic effect of ZnO-NPs in combination with antibiotics on *S. aureus* was characterized using the checkerboard method. The effect of ZnO-NPs on *S. aureus* cell surface hydrophobicity and blood hemolysis was investigated. RT-qPCR was used to investigate the effect of ZnO-NPs on the expression of biofilm related genes (*icaA, icaR* and *sarA*), *katA* and *sigB*. The impact of ZnO-NPs on *S. aureus* pathogenesis was evaluated using mice infection model.

**Results:**

ZnO-NPs exhibited a good antibiofilm activity against *S. aureus*. The findings indicate a synergistic antibiofilm effect of combination between ZnO-NPs and tested antibiotics. ZnO-NPs were capable of decreasing *S. aureus* cell surface hydrophobicity which could account for observed decrease in bacterial biofilm forming capacity. Moreover, ZnO-NPs-treated bacteria exhibited a significant decrease in blood hemolysis relative to control untreated *S. aureus*. The expression of biofilm related genes was significantly repressed in ZnO-NPs treated bacteria as compared to untreated cells. Finally, the effect of ZnO-NPs on *S. aureus* pathogenesis was investigated using mice infection model where ZnO-NPs accelerated healing of wounds in mice as compared to control untreated mice.

**Conclusions:**

Present data support the efficiency of ZnO-NPs as antibiofilm agent in treatment of *S. aureus* infections. This study recommends the incorporation of ZnO-NPs as adjuvant with other antibiotics targeting *S. aureus* based on the promising findings obtained herein in order to control infection with this pathogen.

**Supplementary Information:**

The online version contains supplementary material available at 10.1186/s12866-022-02658-z.

## Introduction

*Staphylococcus aureus* is a Gram-positive opportunistic pathogen that causes a variety of infections both in nosocomial and community settings*. S. aureus* is a causative agent of wide range of illnesses due to its higher capability to colonize and survive within different host tissues. For instance, *S. aureus* causes soft tissue infections, endocarditis, osteomyelitis and urinary tract infections. In addition, *S. aureus* is responsible for several syndromes caused by secretion of exotoxins and enterotoxins including food poisoning and scalded skin syndromes. *S. aureus* expresses many potential virulence factors that promote colonization of host tissues including hemolysins and exotoxins [1].

Bacterial biofilm could be defined as ability of bacterial cells to adhere to various surfaces and the formation a matrix-encased community of cells. Bacterial biofilms could be influenced by various environmental factors including nutrient and oxygen availability. Biofilm formation markedly contributes to *S. aureus* pathogenesis in host. Bacteria within biofilms display a higher resistance to antimicrobial agents including antibiotics. Moreover, bacterial biofilm plays a role in evasion of host immune defenses leading to a difficulty of pathogen removal. The increased biofilm associated staphylococcal infections requires the introducing of effective antibacterial treatment policies [2]. The increased bacterial resistance within biofilms to both antimicrobial agents and host defense is considered the major problem associated with *S. aureus* biofilms. The biofilm polysaccharide matrix has been shown to play an important role in this bacterial resistance and acts as a barrier that hinders the diffusion of antimicrobial agents. Furthermore, differences in nutrient and oxygen availability within biofilm lead to variations in both bacterial growth rate and metabolic activity. As a result, cell division occurs at much lower rates producing persister cells which are highly tolerant to various antimicrobial agents [3].

Nanotechnology has been previously shown to be a promising approach for treatment of biofilm-related infections. Many advantages have been reported for nanoparticles (NPs) as drug delivery systems that maintain sustained drug release and minimize the side effects of used drugs. Furthermore, various NPs are being widely studied for their potential antimicrobial effects. For example, metallic oxide NPs such as zinc oxide (ZnO-NPs) and copper oxide (CuO-NPs) are characterized by low cytotoxicity and higher capacity to function as drug delivery systems. It has been shown that both the antibacterial and antibiofilm properties of nanomaterials could be largely affected by many factors such as shape and surface charge [4, 5].

Importantly, ZnO-NPs have been reported to exhibit many merits such as higher solubility, bioavailability and biocompatibility. Therefore, ZnO-NPs can mimic the biomolecules activity, localize in many body systems and maintain cellular homoeostasis [6]. Compared with other nanomaterials, ZnO-NPs are relatively less toxic, comparatively inexpensive and therefore could be applied in various applications as anticancer, antibacterial as well as anti-inflammation and diabetes treatment [7]. Furthermore, ZnO-NPs are characterized by chemical stability, strong adsorption ability in addition to their catalytic efficiency [8, 9]. On the other hand, other nanomaterials such as silver NPs and iron oxide NPs have been shown to exhibit serious disadvantages such as cytotoxicity that could limit their use in medicine. In addition, silver NPs induce the release of proinflammatory cytokines and production of reactive oxygen species (ROS) and nitric oxide which affect the central nervous system causing neuroinflammation. Moreover, silver NPs have cytotoxic effects on osteoclasts, osteoblasts, cardiovascular and respiratory systems which could restrict their use in biomedical applications [10]. Despite of the advantageous characteristics of gold NPs such as biocompatibility and low toxicity, it is considered as non-biodegradable which may lead to gold accumulation inside the body and possible interaction with immune cells [11]. Finally, silica NPs have been recently used in the treatment of bacterial infection [12]. However, silica NPs exhibit high density of silanol groups on their surface that could interact with the phospholipids of red blood cells leading to hemolysis which requires surface modification in order to be safely used. In addition, the lower stability and formation of aggregates are another problem that could affect the usage of silica NPs in medicine [13]. Detailed comparison between ZnO-NPs and other nanomaterials based on their advantages and disadvantages is represented in the Supplementary Table S1.

Current study aims to investigate the antibacterial and antibiofilm potential of ZnO-NPs against *S. aureus*. The influence of ZnO-NPs on both *S. aureus* susceptibility to antibiotics and host pathogenesis will be investigated herein. Importantly, in addition to *S. aureus* reference strain, the antibiofilm activity of ZnO-NPs on *S. aureus* clinical isolates from various sources will be evaluated herein. Investigating the influence of ZnO-NPs on biofilm formation by clinical *S. aureus* isolates would be important to understand how environment could affect bacterial response to antibiofilm agents. Furthermore, present study is performed in order to characterize the antibacterial and antibiofilm mechanisms of ZnO-NPs. For instance, ZnO-NPs effect on both *S. aureus* cell surface hydrophobicity and hemolytic activity will be assessed. The findings of this study would expand understanding of *S. aureus* pathogenesis and consequently help us control infections caused by this opportunistic pathogen.

## Materials and methods

### Isolation and identification of *S. aureus*

Clinical *S. aureus* isolates were provided by the clinical laboratories of Zagazig University Hospitals and El-Ahrar Educational Hospital in Zagazig, Egypt with no direct involvement of patients in the study. *S. aureus* isolates were further identified biochemically. In addition to *S. aureus* clinical isolates, *S. aureus* ATCC 6538 was involved in this study. For long term preservation of *S. aureus* isolates, glycerol (20%) was added to overnight culture of Mueller Hinton (MH) broth and kept at -80 °C.

### Determination of antibiotic susceptibility *S. aureus* isolates by disc diffusion method

Antimicrobial susceptibility testing was done by Kirby-Bauer standard disc diffusion method against all clinical *S. aureus* isolates and ATCC 6538 according to CLSI (2018) guidelines. The antibiotic discs used in this study include methicillin, ceftriaxone and cefotaxime, tetracycline, ciprofloxacin, gentamicin, erythromycin, azithromycin, linezolid, vancomycin, clindamycin, chloramphenicol and sulfamethoxazole-trimethoprim. The inhibition zones diameters were measured in millimeter (mm) and *S. aureus* susceptibility to antibiotic was interpreted as susceptible (S), intermediate (I) and resistant (R) according to interpretative criteria for antimicrobial susceptibility testing recommended by CLSI (2018) [14].

### Determination of minimum inhibitory concentration (MIC) of tested antibiotics and ZnO-NPs

ZnO-NPs were purchased from (Sigma-Aldrich, Germany) with particle size of 50 nm and surface area of > 10.8 m^2^/g. The MICs of both antibiotics and ZnO-NPs against *S. aureus* ATCC 6538 and clinical isolates were determined by the broth microdilution method according to CLSI (2018). The range of molar concentrations of ZnO-NPs and antibiotics used in MIC assay was as follow; ZnO-NPs (6.29 to 0.01 mM); gentamicin (2.14—0.52 × 10^–3^ mM); ciprofloxacin (3.09—0.18 × 10^–3^ mM); clindamycin (2.4—1 × 10^–5^ mM); chloramphenicol (0.396—0.39 × 10^–3^ mM); cefotaxime (4.5—0.27 × 10^–3^ mM) and azithromycin (1.36—1.7 × 10^–4^ mM). The MIC of selected antibiotics was defined as the lowest concentration of antibiotic that inhibits bacterial growth and no visible growth is observed as compared with both a positive control (culture broth containing bacteria only that should appear turbid) and a negative control (culture broth without bacteria that should remain clear) [14]. In addition, the half maximal inhibitory concentration (IC_50_) of ZnO-NPs against *S. aureus* was determined according to Namvar et al. [15]. The experiment was performed in triplicate and the IC_50_ was calculated by plotting the percentage of growth inhibition to a normal dose–response-inhibition curve.

### Quantitative assessment of biofilm formation by spectrophotometry

The ability of *S. aureus* ATCC 6538 and clinical isolates to form biofilm was tested according to Stepanović *et al.* [16] with some modifications. A 1:100 dilutions of *S. aureus* overnight culture were prepared in Trypticase soy (TS) broth supplemented with 1% glucose (TSB_1%GLU_). Aliquots of 200 µL/well of the prepared suspensions were added to each well of 96 well microtiter plates and incubated for 48 h at 37 °C. Negative control wells that contained 200 µL/well of TSB only were included in each plate. Formed biofilms were fixed using methanol for 20 min. Fixed biofilms were stained with 1% crystal violet for 15 min. The plates were air dried and the bound dye was dissolved by 33% glacial acetic acid. The optical densities were measured spectrophotometrically at 570 nm using Bio-Tek synergy HT microplate reader, USA. Measurements were performed in triplicates and the experiment was repeated three times. The cut-off optical density (ODc) was calculated as three times standard deviations above the mean OD of the negative control. The tested strains were classified into: non-biofilm producer (OD ≤ ODc), weak biofilm producer (OD > ODc, but ≤ 2 × ODc), moderate biofilm producer (OD > 2 × ODc, but ≤ 4 × ODc) and strong biofilm producer (OD > 4 × ODc) [16].

### Antibiotic susceptibility testing for bacterial biofilm

The susceptibility of biofilm formed by *S. aureus* ATCC 6538 and clinical isolates to antibiotics was assayed according to Sabino *et al.* [17] in 96-wells polystyrene microtiter plates. A 1:100 dilution of *S. aureus* overnight culture with turbidity matching that of 0.5 McFarland standards were made in TSB_1%GLU_, then 100 µL aliquots was transferred to wells of microtiter plates. The plates were incubated for 48 h at 37 °C for biofilm formation. Wells were washed thrice with PBS under aseptic conditions to remove unattached bacteria and dried in an inverted position. Two-fold serial dilutions of the respective antibiotic in MH broth were added into the dried wells with established biofilms. The microtiter plates were incubated for 24 h at 37 °C, and minimum biofilm inhibitory concentration (MBIC) was determined. The experiment was performed in triplicate [17].

### Assay of antibiofilm ZnO-NPs by microtiter plate method

The antibiofilm potential of ZnO-NPs against both *S. aureus* ATCC 6538 and clinical isolates was characterized according to Basumatari *et al*. [18]. Briefly, overnight grown cultures of bacteria were diluted to 1:100 in TSB_1%GLU_. Then aliquots of about 100 µL were added to wells of sterile microtiter plate and incubated for 48 h to allow biofilm formation. The biofilm eradication activity of ZnO-NPs was determined as follows; aliquots of different concentrations of ZnO-NPs; 256, 128 and 64 µg /mL (3.1, 1.57 and 0.78 mM; respectively) were added to plate. Both negative and positive control wells without bacterial culture and without ZnO-NPs respectively were maintained for each isolate. The plate was incubated at 37 °C for 24 h and the influence of ZnO-NPs on bacterial biofilm eradication was assessed as described above. Each assay was performed in triplicate and percentage of biofilm inhibition was determined as follows [18]:

$$\%\;\mathrm o\mathrm f\;\mathrm b\mathrm i\mathrm o\mathrm f\mathrm i\mathrm l\mathrm m\;\mathrm i\mathrm n\mathrm h\mathrm i\mathrm b\mathrm i\mathrm t\mathrm i\mathrm o\mathrm n\;=\frac{\mathrm{OD}\;\mathrm{in}\;\mathrm{control}-\mathrm{OD}\;\mathrm{in}\;\mathrm{treatment}}{\mathrm{OD}\;\mathrm{in}\;\mathrm{control}}\times100$$

### Characterization of the synergistic effect of ZnO-NPs in combination with antibiotics on inhibition of *S. aureus* biofilm formation

The effect of combination between ZnO-NPs and tested antibiotics (AB) on inhibition of *S. aureus* ATCC 6538 biofilm formation was determined by test tube method as described by Ashajyothi *et al*. [19]. Four separate test tubes were used as follow; tube 1 contained bacterial culture + TSB_1%GLU_ as control, tube 2 contained bacterial culture + TSB_1%GLU_ + corresponding antibiotic, tube 3 contained bacterial culture + TSB_1%GLU_ + ZnO-NPs and tube 4 contained bacterial culture + TSB_1%GLU_ + antibiotic + ZnO-NPs. All tubes were incubated for 48 h at 37 °C. Next, each tube was washed with PBS and biofilm formation was characterized as described above. Percentage of synergistic effect of ZnO-NPs on biofilm inhibition by antibiotics was calculated by following equation [19].

$$\%\;\mathrm{of}\;\mathrm{synergetic}\;\mathrm{effect}\;=\;\frac{\%\mathrm{of}\;\mathrm{inhibition}\;\mathrm{of}\;\left(\mathrm{ZnO}-\mathrm{NPs}\;+\;\mathrm{AB}\right)-\%\;\mathrm{of}\;\mathrm{inhibition}\;\mathrm{of}\;(\mathrm{ZnO}-\mathrm{NPs})}{\%\;\mathrm{of}\;\mathrm{inhibition}\;\mathrm{of}\;\left(\mathrm{ZnO}-\mathrm{NPs}\;+\;\mathrm{AB}\right)}\times\;100$$

### Checkerboard microdilution assay

Checkerboard assay was performed to measure synergy between ZnO-NPs and different antibiotics against *S. aureus* ATCC 6538 by calculating the fractional inhibitory concentration index (FICI) where FIC index (FICI) = FIC of ZnO-NPs + FIC of antibiotic. Synergy was defined when FICI was ≤ 0.5; while additive in which 0.5 ˂ FICI ≤ 1.0; whereas indifferent when the FICI is between 1 and 4 [19]. The range of molar concentrations used in combination assays was as follow; ZnO-NPs (6.29—6 × 10^–3^ mM); gentamicin (16 × 10^–3^—6 × 10^–5^ mM); ciprofloxacin (12 × 10^–3^—9 × 10^–5^ mM); clindamycin (14 × 10^–5^—1 × 10^–5^ mM); chloramphenicol (24 × 10^–3^—5 × 10^–5^ mM); cefotaxime (0.56—1 × 10^–3^ mM) and azithromycin (1.3 × 10^–3^—1.7 × 10^–4^ mM).

### Characterization of *S. *aureus biofilm inhibition by scanning electron microscopy (SEM) and light microscopy

The antibiofilm activity of ZnO-NPs, gentamicin and their combination against *S. aureus* ATCC 6538 was characterized using both scanning electron microscope (SEM) and light microscope. Biofilms were developed on polystyrene discs exactly as described above for control untreated, gentamicin-treated, ZnO-NPs-treated and ZnO-NPs + gentamicin-treated bacteria. Following incubation, discs were washed trice with phosphate buffered saline (PBS) and fixed by 2.5% glutaraldehyde for 2 h. Discs were dried and gold coated before imaging and examined using jeol scanning microscope (JSM- T100, Japan) [20].

### Determination of hydrophobicity index (HI) of ZnO-NPs treated *S. aureus*

Hydrophobicity index (HI) of ZnO-NPs treated *S. aureus* ATCC 6538 cells was done according to Rauf et al. [21]. Briefly, overnight grown bacterial cells were resuspended in MH broth and the optical density was adjusted to 1.0 at a wavelength of 595 nm. A volume of 1 mL toluene was added to cell suspension in a test tube and the tube was vortexed. The biphasic mixture of two phases was allowed to settle for 30 min and the optical density of the aqueous phase was measured. Hydrophobicity index (HI) of microbial cells was determined by using the following equation [21]: ${\text{HI}} \, \text{\% = }\frac{\mathrm{Ai}-\mathrm{Af}}{\mathrm{Ai}}\times 100$**,** where Ai and Af are the initial and final optical densities of the aqueous phase; respectively.

### Haemolysis assay

*In vitro* erythrocyte lysis test was performed according to Rauf *et al.* [21]. Briefly, fresh blood isolated from a healthy sheep was collected in anticoagulant solution (EDTA) and spun at 6000 rpm for 10 min at 4 °C. Both buffy coat and plasma were discarded. Washed erythrocytes were diluted with isotonic buffer (20 mM PBS) to prepare 2% hematocrit. The extent of hemolysis was studied by incubating red blood cells (RBCs) suspension with *S. aureus* ATCC 6538 alone and *S. aureus* with ZnO-NPs at 37 °C overnight. In addition, erythrocytes were incubated with ZnO-NPs alone as a control to confirm that ZnO-NPs have no hemolytic effect on erythrocytes at used concentration. Next, incubated solutions were centrifuged at 6000 rpm for 10 min and the supernatant was analyzed for released hemoglobin and measured at 490 nm. The percentage hemolysis was determined by the following equation [21]:

 $\%\;\mathrm{Haemolysis}\;=\left[\{\mathrm{AbsT}-\;\mathrm{AbsC}\}/\{\mathrm{Abs}(100\%)-\mathrm{AbsC}\}\right]\;\times\;100$ where; AbsT is the absorbance of the supernatant from samples incubated with *S. aureus*, *S. aureus* with ZnO-NPs respectively, AbsC is the absorbance of the supernatant from control (PBS) and Abs 100% is the absorbance in the presence of 0.1% sodium dodecyl sulfate (SDS).

### Total RNA extraction and qRT-PCR

*S. aureus* ATCC 6538 at cell density of 1 × 10^6^ CFU∕mL was cultured in TSB _1%GLU_ with or without 0.78 mM ZnO-NPs (64 µg∕mL) for 48 h. Next, bacterial suspensions were centrifuged at 6000 rpm for 10 min at 4 °C and cell pellets were washed with PBS. RNA was extracted from both treated and untreated bacterial pellets using GeneJET RNA Purification Kit (Thermoscientific, USA) following the manufacturer instructions. Obtained RNA was further purified using a Qiagen RNeasy minikit (Qiagen, Valencia, CA). Following purification, 100 μL of nuclease free water was added to elute RNA and stored at -70 °C until use. The expression of *S. aureus* biofilm-related genes (*icaA, icaR, agrA* and *sarA*), oxidative stress response gene; catalase (*katA*) and the transcription regulator sigma B (*sigB*) was determined in both ZnO-NPs treated and untreated *S. aureus* ATCC 6538 using q RT-PCR. The expression level of each gene was normalized to 16S rRNA, a gene for which the expression levels remained unchanged in both control and treated with ZnO-NPs bacteria. Primers used in this study are listed in Table 1. The analysis was conducted following the protocol described in SensiFAST™ SYBR Hi-ROX One-Step Kit (Bioline, UK) using StepOne Real-Time PCR system (Applied Biosystem, USA). Specific PCR amplification was confirmed by both agarose gel electrophoresis and a melting curve analysis of products according to the manufacturer recommendation. Relative gene expression was calculated by the 2^−∆∆*CT*^ method [22].

**Table 1:** Primers used in qRT-PCR

**Gene name**	**Primer sequence (5‵- 3‵)**
*icaA* (F)	CTGGCGCAGTCAATACTATTTCGGGTGTCT
*icaA* (R)	GACCTCCCAATGTTTCTGGAACCAACATCC
*icaR* (F)	TGCTTTCAAATACCAACTTTCAAGA
*icaR* (R)	ACGTTCAATTATCTAATACGCCTG
*sigB* (F)	AAGTGATTCGTAAGGACGTCT
*sigB* (R)	TCGATAACTATAACCAAAGCCT
*agr* (F)	TGATAATCCTTATGAGGTGCTT
*agr* (R)	CACTGTGACTCGTAACGAAAA
*sarA* (F)	CAAACAACCACAAGTTGTTAAAGC
*sarA* (R)	TGTTTGCTTCAGTGATTCGTTT
*katA* (F)	AAAGGTTCTGGTGCATTTGG
*katA* (R)	AACGCAAATCCTCGAATGTC
*16s RNA* (F)	ACTCCTACGGGAGGCAGCAG
*16s RNA* (R)	ATTACCGCGGCTGCTGG

*F* Forward, *R* Reverse

### Determination of ZnO-NPs effect on staphylococcus biofilm formation on urinary catheter

The inhibitory efficacy of ZnO-NPs on staphylococcal biofilms on catheters was determined *in vitro* [23]. An overnight culture of *S. aureus* ATCC 6538 was diluted 1:100 in TSB_1%GLU_. Sterile 1 cm long segments of Foley catheter were immersed in 5 mL culture tube containing approximately 10^6^ CFU∕mL of *S. aureus* ATCC 6538 and another tube containing sub-MIC of ZnO-NPs (64 µg∕mL; 0.78 mM). Cultures with catheter pieces were incubated at 37 °C for 48 h. Following incubation, catheter pieces were removed and rinsed twice in PBS to remove the adhering planktonic cells. Next, segments were transferred to another tube containing 5 mL PBS in order to extract biofilm bacterial cell from catheter. Tubes were vortexed for 1 min then sonicated for 15 min and vortexed again for 1 min. Serial dilutions of bacteria were made in sterile PBS, plated onto mannitol salt agar and incubated for 24 h at 37 °C for viable counting.

## *In vivo* mice infection

The influence of ZnO-NPs on *S. aureus* ATCC 6538 pathogenesis was characterized using mice infection model according to [24]. The animal study was approved by the institutional animal care and used committee, Zagazig University (ZU-IACUC) with approval number ZUIACUC/3/F/159/2019. Thirty-six healthy albino mice were used in this study and randomly divided into 6 groups (6 mice each). In addition to the positive control group (*S. aureus*-inoculated mice) and negative control groups (PBS-inoculated mice and uninfected mice), other three groups were included; ZnO-NPs treated mice group, gentamicin-treated mice group and ZnO-NPs + gentamicin treated mice group. Mice were anesthetized with thiopental through intraperitonial injection of 100 µL of 40 mg Kg^−1^. The back of mice was disinfected with 70% alcohol and hair was shaved. Full thickness skin wounds were created by sterile surgical scissor and immediately inoculated intradermally with *S. aureus* suspension at density (2.5 × 10^7^ CFU∕mL) to each wound site. Wounds were treated intradermally with gentamicin, ZnO-NPs or a combination of gentamicin + ZnO-NPs. The progression of wound healing in different mice groups was assessed every 3 days following bacterial inoculation (day zero). Results were taken by capturing representative images for wound healing in mice in different groups on days 0, 3, 6, 10, and 14 following treatment using a camera (Sony Ciyber Shot, Dsc w80). In addition, the wound contraction percentage (%) was determined by calculating the wound area (mm^2^) at the analysis day and compared with the initial wound area (mm^2^) using the following formula [25]: Wound contraction (%) = $\frac{\mathrm{A}0-\mathrm{At}}{\mathrm{A}0} \times 100$ where; A0 is the initial wound area in mm^2^ and At is the wound area at the time of image capturing in mm^2^.

## Results

### Isolation and identification of *S. aureus* isolates

A total of 103 *S. aureus* isolates were involved in current study. *S. aureus* isolates were from different clinical sources including wound infections, urinary tract infections, respiratory tract infections, burn, eye and ear infections. Bacterial isolates were confirmed as *S. aureus* based on their biochemical characteristics.

### Determination of antibiotic susceptibility of* S. aureus* isolates by disc diffusion method

*S. aureus* isolates showed varying resistance patterns to different antibiotics (Supplementary Table S2). All *S. aureus* clinical isolates were resistant toward methicillin. High bacterial resistance was detected against cefotaxime and ceftriaxone (92.2% each) and tetracycline (63.1%). Intermediate bacterial resistance was observed against gentamicin (47.5%), erythromycin (31.1%) and azithromycin (30.1%). Low bacterial resistance was recorded against chloramphenicol, ciprofloxacin, clindamycin and sulphamethoxazole-trimethoprim (22.3%, 20.4%, 6.7% and 5.8%; respectively). All *S. aureus* clinical isolates were sensitive to both linezolid and vancomycin. Many *S. aureus* isolates (64%) were multi-drug resistant (MDR), which could be defined as resistance to at least one member in three or more different categories of antibiotics.

### Determination of MIC of tested antibiotics and ZnO-NPs against *S. aureus* isolates

**Fig. 1:**
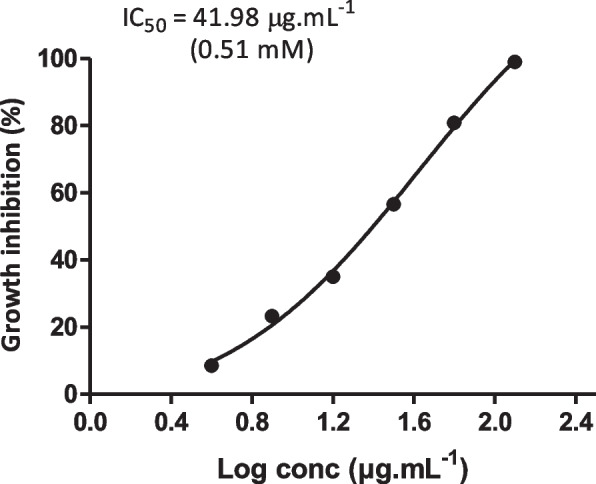
Determination of the half maximal inhibitory concentration (IC_50_) of ZnO-NPs against *S. aureus* ATCC 6538. Data shown represent the mean ± standard error from triplicate experiments. IC_50_ value was calculated from a non-linear regression model

The MICs of tested antibiotics and ZnO-NPs were determined against both *S. aureus* ATCC 6538 and twenty clinical isolates recovered from different sources and exhibited variable antibiotic resistance profiles by disc diffusion method. The MICs were determined by the broth microdilution method according to the CLSI (2018) and results are represented in (Supplementary Table S3). In addition, the half maximal inhibitory concentration (IC_50_) of ZnO-NPs against *S. aureus* was determined and found to be 0.51 mM (Fig. 1) indicating that ZnO-NPs exhibit a potent antibacterial activity.

## Quantitative assessment of *S. aureus* biofilm formation

**Fig. 2:**
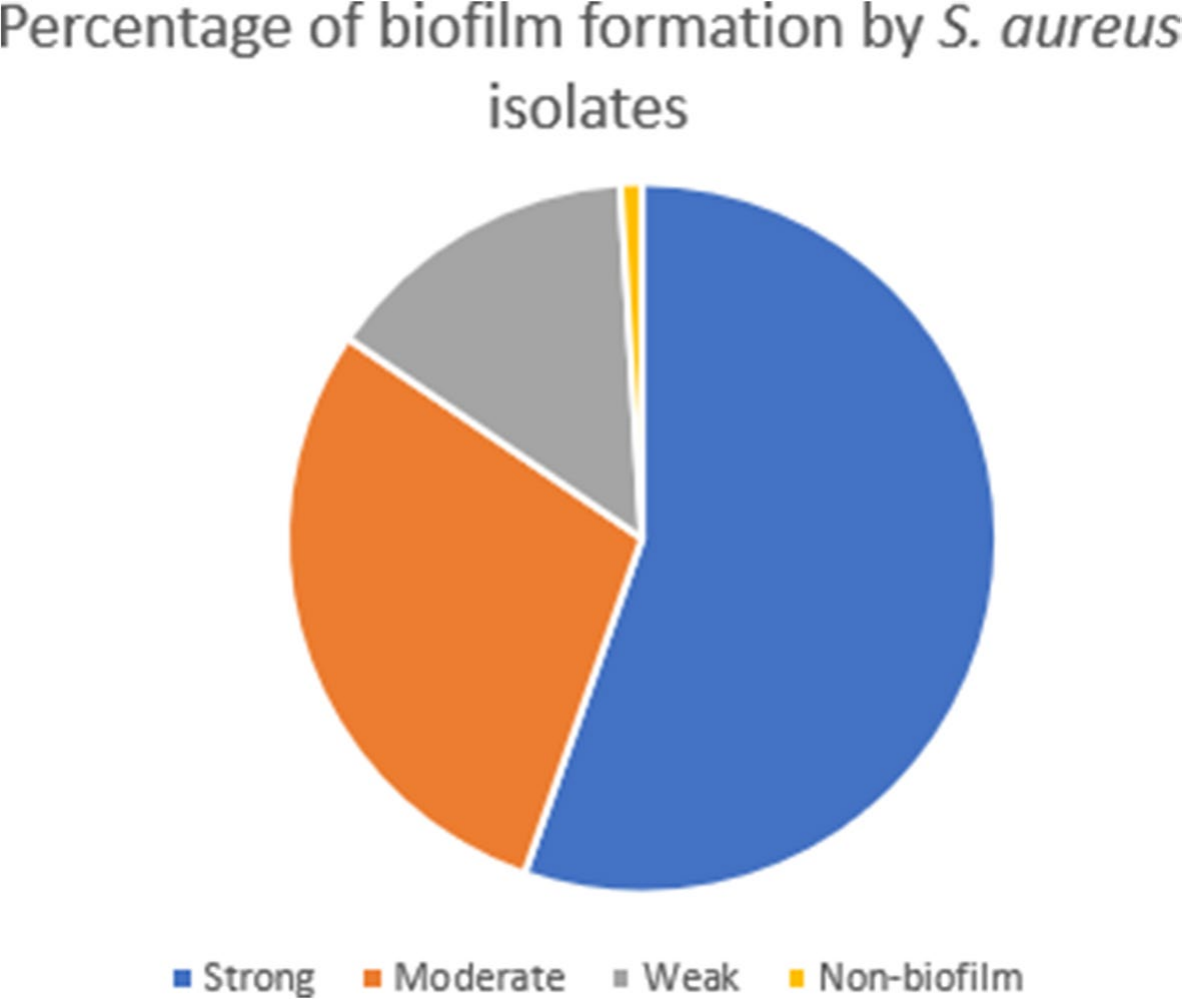
Quantitative assessment of *S. aureus* isolates biofilm formation. Bacterial biofilms were quantified by crystal violet assay. Optical density (OD) was measured spectrophotometrically at 570 nm. *S. aureus* isolates were classified according to biofilm forming capacity into strong, moderate, weak and non-biofilm forming

*S. aureus* ATCC 6538 and clinical isolates were screened for biofilm formation spectrophotometrically at 570 nm. Bacterial isolates were divided into four categories according to their capacity to form biofilm (Fig. 2). These four categories include; non-biofilm forming (OD ≤ 0.163), weak biofilm forming (OD ˃ 0.163, but ≤ 0.326), moderate biofilm forming (OD ˃ 0.326, but ≤ 0.652), strong biofilm forming (OD ˃ 0.652).

### Antibiotic susceptibility testing for bacterial biofilm

The antibiotic susceptibility of biofilm formed by *S. aureus* ATCC 6538 and clinical isolates against different antibiotics was characterized by determining the MBIC. Results are presented in Table 2. All MBIC values were much higher than respective MIC values for the bacterial isolates tested.

**Table 2:** Minimum biofilm inhibitory concentrations (MBICs) of antibiotics against *S. aureus*

**Isolate NO**	**Gentamicin (µg/mL)**		**Ciprofloxacin** **(µg/mL)**		**Clindamycin** **(µg/mL)**		**Chloramphenicol** **(µg/mL)**		**Cefotaxime** **(µg/mL)**		**Azithromycin** **(µg/mL)**	
	**MIC**	**MBIC**	**MIC**	**MBIC**	**MIC**	**MBIC**	**MIC**	**MBIC**	**MIC**	**MBIC**	**MIC**	**MBIC**
***S. aureus* ATCC 6538**	4	32	2	64	0.03	32	4	8	128	8192	0.5	512
**1B**	128	512	2	64	0.06	2	16	128	16	64	4	256
**2B**	32	64	0.125	64	< .003	16	4	16	0.125	4	0.25	128
**24B**	1024	˃4096	128	2048	512	2048	128	1024	2048	8192	> 1024	˃8192
**38B**	1024	4096	64	256	8	64	64	128	1024	4096	> 1024	˃8192
**41B**	2	16	1	128	< .003	32	4	16	0.125	128	1	256
**42B**	64	1024	0.25	128	0.25	32	0.5	256	0.125	64	1	128
**44B**	8	32	1	32	0.25	32	4	8	64	512	1	256
**48U**	4	16	8	64	0.03	32	4	8	256	4096	64	8192
**55EY**	64	256	0.25	64	0.015	32	4	16	0.125	8	0.5	256
**62EN**	8	16	16	64	0.03	2	8	32	32	128	32	8192
**63EN**	8	16	16	256	0.5	1	32	128	128	2048	32	256
**68EA**	0.5	256	32	128	< .003	64	8	64	0.5	2048	0.5	1024
**82W**	2	8	0.5	128	0.015	32	8	16	16	64	0.5	256
**83W**	1	16	0.25	64	0.06	32	8	32	0.5	256	2	256
**91W**	8	32	1024	4096	256	1024	16	32	1024	2048	512	8192
**97W**	1024	4096	32	1024	256	1024	64	128	1024	4096	> 1024	8192
**99W**	256	8192	64	512	4	4096	128	512	1024	4096	32	˃8192
**101W**	1024	˃4096	64	1024	1024	2048	64	1024	1024	8192	> 1024	8192

*B* Burn, *U* Urine, *EY* Eye, *EN* Endotracheal aspirate, *EA* Ear, *W* Wound

### Characterization of antibiofilm activity of ZnO-NPs by microtiter plate method

Antibiofilm activity of ZnO-NPs against both *S. aureus* ATCC 6538 and four *S. aureus* clinical isolates from different sources was characterized by microtiter plate method and percentages of biofilm inhibition are presented in Table 3. ZnO-NPs significantly reduced the biofilm biomass of *S. aureus* in a dose-dependent manner and percentages of biofilm reduction ranges from 29.4% to 50.4%.

**Table 3:** Percentage of biofilm inhibition by ZnO-NPs

**Isolate No**	**ZnO-NPs concentration (µg/mL)/ Percentage reduction of biofilm formation (%)**		
	**256 µg/mL**	**128 µg/mL**	**64 µg/mL**
***S. aureus* ATCC 6538**	50.4	37.8	29.4
**42B**	45.2	25.4	0
**62EN**	10.3	8.6	0
**68EA**	13.3	0	0
**99W**	35.7	25.9	20.2

*B* Burn, *EN* Endotracheal aspirate, *EA* Ear, *W* Wound

### Characterization of effect of combination between ZnO-NPs and antibiotics on *S. aureus* biofilm formation

**Fig. 3:**
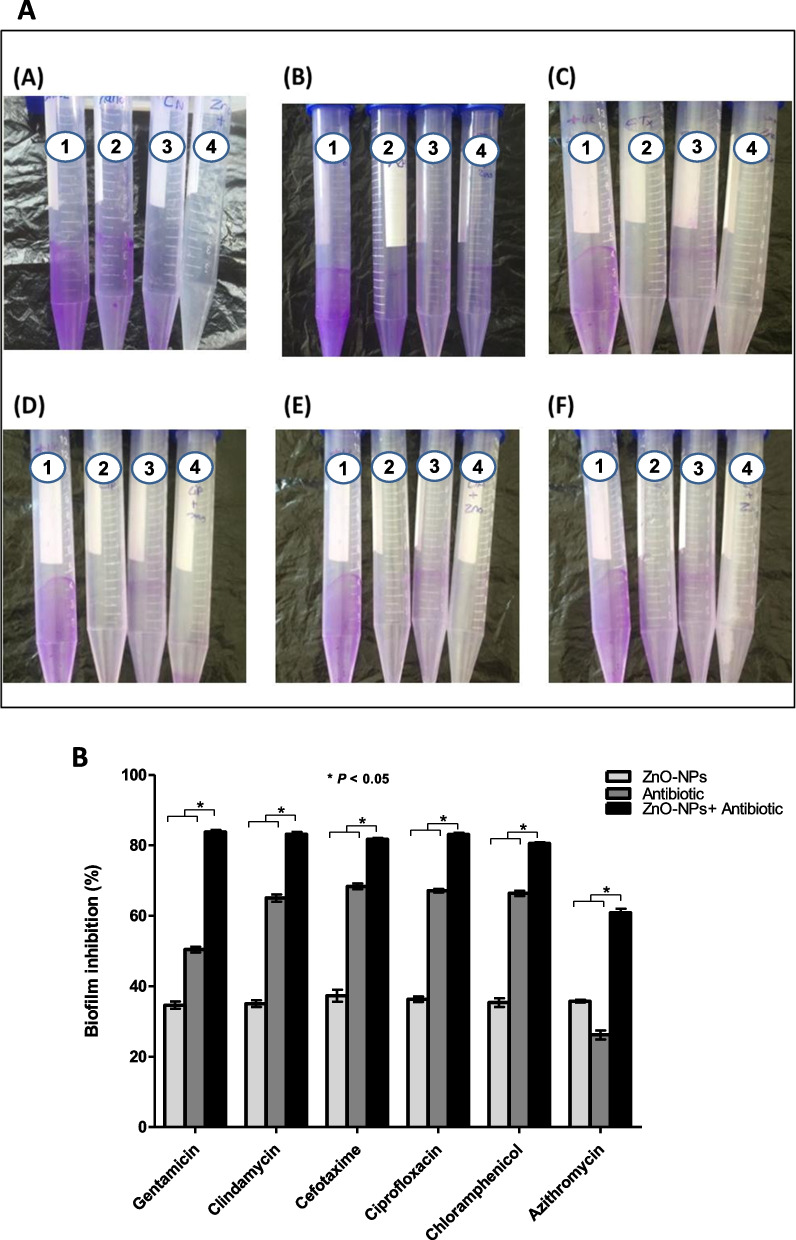
**A** Antibiofilm assay by test tube method. The first tube in all images represents a positive control. The second and third tubes represent biofilm formed by bacterial cells in presence of antibiotic and ZnO-NPs; respectively. The fourth tube represents the biofilm formed by bacterial cells in presence of a combination of both antibiotic and ZnO-NPs. A; Gentamicin, B; Azithromycin, C; Cefotaxime, D; Ciprofloxacin, E; Clindamycin, and F; Chloramphenicol. **B** A combination between ZnO-NPs and antibiotics reduces *S. aureus* biofilm formation. Bacteria biofilms were stained with 1% crystal violet and percentage of biofilm inhibition was analyzed spectrophotometrically. Results are expressed as mean ± SE of three independent experiments. * P < 0.05 was considered significant using 2way ANOVA test

The synergistic effect of combination between ZnO-NPs and tested antibiotics on biofilm inhibition against *S. aureus* ATCC 6538 was investigated by the test tube method (Fig. 3A). Results show that ZnO-NPs alone were capable of inhibiting biofilm formation by 34–37%. However, combination of ZnO-NPs and various antibiotics inhibited biofilm formation by 65–85% (Fig. 3B).

### Characterization of synergy between ZnO-NPs and antibiotics by checkerboard microdilution assay

The synergistic potential of ZnO-NPs with commonly used antibiotics; gentamicin, ciprofloxacin, clindamycin, chloramphenicol, cefotaxime and azithromycin to inhibit planktonic *S. aureus* ATCC 6538 was determined. Synergy was studied by the checkerboard method by determining the FICI (fractional inhibitory concentration index). Importantly, the MICs values for tested antibiotics were reduced when combined with ZnO-NPs giving FIC values ≤ 0.5 indicating a synergistic effect (Table 4). The combination MICs of gentamicin, ciprofloxacin, chloramphenicol, cefotaxime, clindamycin and azithromycin on the *S. aureus* ATCC 6538 dropped to 0.25, 0.125, 0.125, 4, 0.003 and 0.125 µg/mL in contrast to the individual MICs of 4, 2, 4, 128, 0.03 and 0.05 µg/mL, respectively. All tested antibiotics in combination with ZnO-NPs showed a synergistic effect with FICIs ranging from 0.045 to 0.312.

**Table 4:** Fractional inhibitory concentration index (FICI) of ZnO-NPs and tested antibiotics against *S. aureus* ATCC 6538

**Agent**	**FIC**	**FICI**	**Interpretation**
**ZnO-NPs**	0.015	0.075	S
**Gentamicin**	0.06		
**ZnO-NPs**	0.062	0.122	S
**Ciprofloxacin**	0.06		
**ZnO-NPs**	0.25	0.28	S
**Chloramphenicol**	0.03		
**ZnO-NPs**	0.015	0.045	S
**Cefotaxime**	0.03		
**ZnO-NPs**	0.062	0.162	S
**Clindamycin**	0.1		
**ZnO-NPs**	0.062	0.312	S
**Azithromycin**	0.25		

*FIC* Fractional inhibitory concentration, *FICI* Fractional inhibitory concentration index, *S* Synergism

### Microscopical characterization of ZnO-NPs-induced inhibition of *S. aureus* biofilm formation

**Fig. 4:**
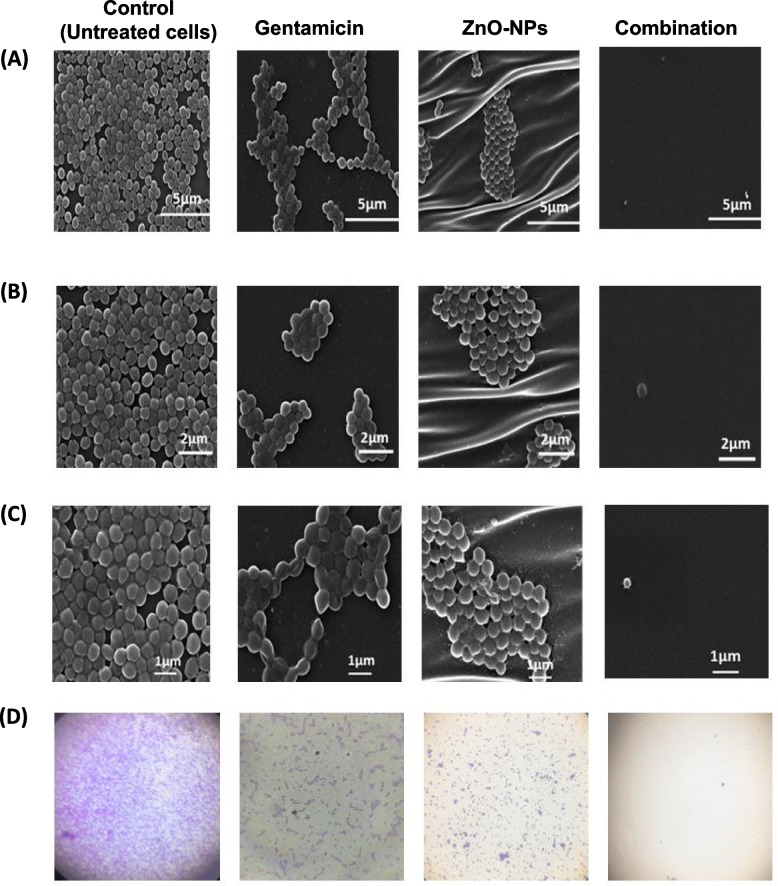
Microscopic visualization of antibiofilm activity of ZnO-NPs, gentamicin and their combination against *S. aureus*. Scanning electron micrographs at various magnifications; **A**) 5000X, **B**) 7500X and **C**) 10000X. **D**) Light microscope images (100X magnification)

The effect of ZnO-NPs on *S. aureus* biofilm was further characterized using both SEM and light microscopy. *S. aureus* biofilm was allowed to develop on the surface polystyrene discs in the presence of 0.78 mM ZnO-NPs (64 µg/mL), gentamicin (2 µg/mL) as well as their combination and compared to untreated cells. The morphology of *S. aureus* biofilm architecture formed was visualized microscopically under various magnifications. Under 5000 × and 7500 × magnification, SEM analysis revealed a thick, dense and fully established biofilm consisting of multi-layered bacterial cells for untreated cells (Fig. 4A & B). Upon treatment with ZnO-NPs or gentamicin, biofilm production was highly disrupted and a minimum biofilm mass was detected on polystyrene discs. Bacteria appeared as a monolayer of dispersed cells scattered on the surface. At higher magnification (10000x), untreated *S. aureus* appeared as large aggregates of cells in overlapped layers (Fig. 4C). In contrast, bacteria treated with ZnO-NPs or gentamicin presented as a uniform layer of cells with minimal clumping. Treatment with a combination of ZnO-NPs and gentamicin significantly lowered bacterial biofilm formation where there was a marked disruption of biofilm production and a negligible biofilm mass was detected (Fig. 4 A, B & C). The results obtained by SEM analysis were in agreement with those revealed by light microscopy (Fig. 4D). Microscopical images validate the data from the crystal-violet biofilm assay indicating a potential role of ZnO-NPs in inhibition of *S. aureus* biofilm formation.

### Evaluation of hydrophobicity index

**Fig. 5:**
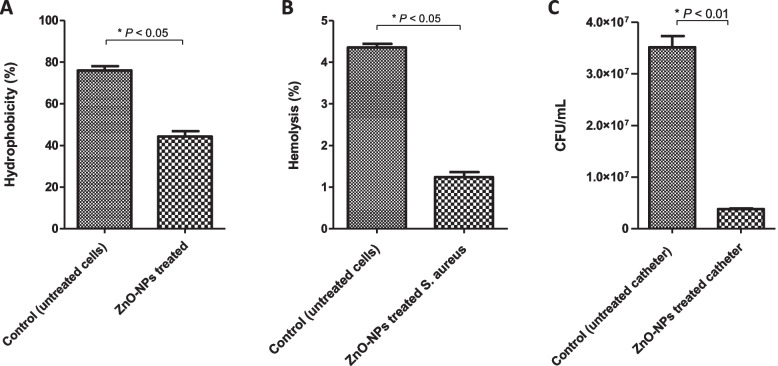
**A** ZnO-NPs reduced hydrophobicity index of *S. aureus*. Bacterial cells were grown overnight in presence and absence of ZnO-NPs. Bacterial density adjusted to 1.0 ± 0.01, then 1 ml of toluene was added to bacterial suspension. After that optical density of aqueous phase was measured. **B** ZnO-NPs inhibited *S. aureus*-induced haemolysis. RBCs were incubated with *S. aureus* alone (control) or *S. aureus* treated with ZnO-NPs. Percentages of haemolysis were measured. **C** ZnO-NPs decreased *S. aureus* biofilm formation on urinary catheter. Catheter segments were immersed in *S. aureus* culture in presence or absence of sub-MIC of ZnO-NPs. Following incubation for 48 h, bacterial counts on the surface of catheter were determined after serial dilution in PBS and plating on mannitol salt agar. The total number of biofilm cells of ZnO-NPs treated catheters was significantly (*P < 0.05) less than untreated catheter. All results are expressed as means ± SE of three independent experiments using Student’s *t* test

Changes in cell surface hydrophobicity of *S. aureus* ATCC 6538 cells following exposure to sub-MIC of ZnO-NPs 64 µg/mL (0.78 mM) were investigated. Higher hydrophobicity of bacteria means increased cell adherence to various surfaces that enhances biofilm formation. As shown in Fig. 5A, *S. aureus* surface hydrophobicity was significantly (*P* < 0.05) affected upon treatment with sub-MIC of ZnO-NPs. On contrast to untreated bacteria that showed higher surface hydrophobicity (76% ± 1.2) the cell surface hydrophobicity of ZnO-NPs-treated cells was significantly reduced (44.33% ± 1.5). These results clearly indicate that ZnO-NPs were capable of altering *S. aureus* cell surface hydrophobicity and consequently the bacterial biofilm forming capacity could be decreased.

### Haemolysis assay

Pre-incubation of *S. aureus* with sub-MIC of ZnO-NPs decreased the *S. aureus*-induced RBCs lysis. As shown in Fig. 5B, ZnO-NPs treated bacterial cells exhibited a significant decrease in hemolysis capacity (1.24% ± 0.08) relative to control untreated bacteria (4.35% ± 0.12). Of note that, no hemolysis was observed when RBCs incubated with ZnO-NPs alone at used concentration (0.78 mM). These findings indicate that prior treatment of *S. aureus* by ZnO-NPs interferes with the hemolytic capability of *S. aureus*.

### *In vitro* inhibition of *S. *aureus biofilm formation on urinary catheter by ZnO-NPs

The capacity of ZnO-NPs to inhibit staphylococcal biofilms on the surface of urinary catheter was evaluated. Treatment with ZnO-NPs (0.78 mM) showed a significant reduction in biofilm mass and bacterial count (Fig. 5C). The bacterial cell count on the surface of ZnO-NPs treated catheter (3.50 × 10^4^ ± 2.1 × 10^6^ CFU/mL) was markedly lower than that (3.5 × 10^7^ ± 1.5 × 10^5^ CFU/mL) on the surface of control untreated catheter. These results clearly indicate that ZnO-NPs possess a potent antibiofilm activity on *S. aureus* biofilms growing on catheters.

### Influence of ZnO-NPs on expression of *S. aureus* biofilm and oxidative stress genes

**Fig. 6:**
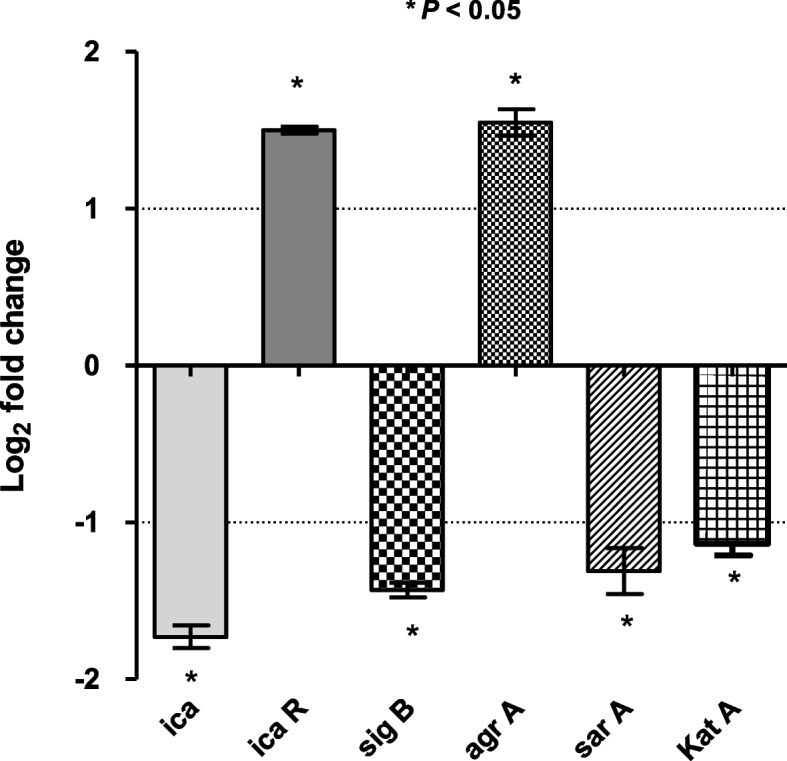
Characterization of influence of ZnO-NPs on the expression of *S. aureus* biofilm and oxidative stress genes by RT- qPCR. The expression levels of *icaA, icaR, agrA, sarA*, *katA* and *sigB* in both ZnO-NPs treated and untreated *S. aureus* ATCC 6538 were analyzed by qRT-PCR. The expression level of each gene was normalized to 16S *rRNA* and expression foldchange was calculated by the 2^−∆∆CT^ method. The data shown are the means ± standard errors from three biological experiments with three technical replicates each. * P < 0.05was considered significant

The expression levels of biofilm genes, catalase; *katA* and *sigB* were determined in both ZnO-NPs treated and untreated *S. aureus* ATCC 6538 by qRT-PCR. The expression of *icaA, sarA, katA* and *sigB* was significantly repressed in ZnO-NPs treated bacteria as compared to untreated cells. On the other hand, the expression of *icaR* and *agrA* that plays a role in biofilm formation was significantly induced in ZnO-NPs treated *S. aureus* relative to untreated cells (Fig. 6). These results indicate that exposure of *S. aureus* to ZnO-NPs negatively affects the expression of genes involved in both biofilm formation and oxidative stress response.

### *In vivo* characterization of the influence of ZnO-NPs on *S. aureus* pathogenesis using mice infection models

**Fig. 7:**
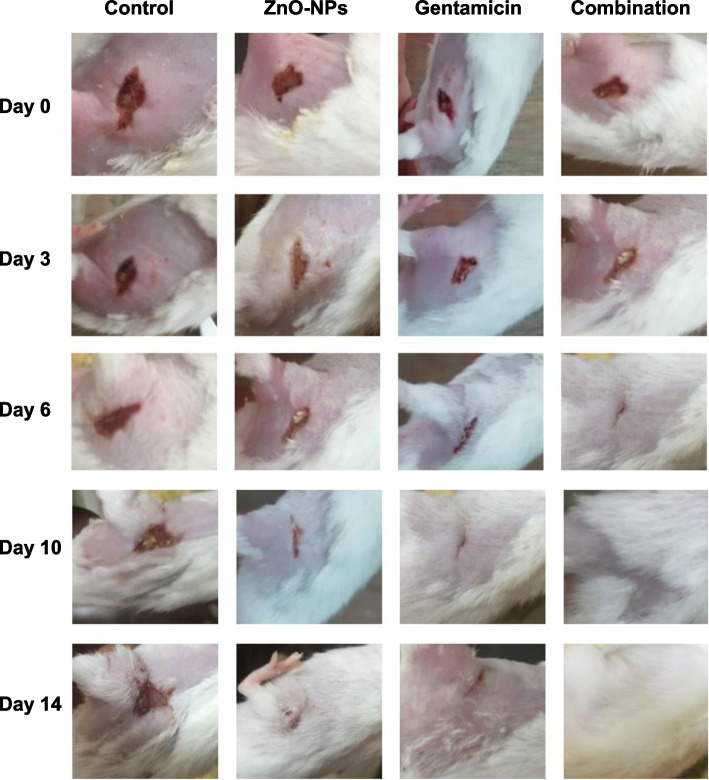
ZnO-NPs improved healing of full thickness wounds in mice. Mice were anesthetized and hair on their backs was shaved. Skin wounds were created and inoculated with *S. aureus* (2.5 × 10^7^ CFU∕mL) at wound site. Wounds were treated with gentamicin, ZnO-NPs or a combination of both. Progression of wound healing was monitored and images of skin wounds on mice from various groups were taken on day 0, 3, 6, 10, and 14 after creation of wound and treatment and represented. Treatment of mice with both ZnO-NPs and gentamicin together accelerates wound healing relative to control untreated mice and those treated with ZnO-NPs or gentamicin alone

The impact of ZnO-NPs on *S. aureus* pathogenesis was further characterized in this study. Importantly, wounds on mice in control groups, both untreated and PBS-treated mice, did not heal completely over the experiment course. Skin wounds in mice treated with ZnO-NPs or gentamicin alone needed more time to heal and did not show complete healing until day 14 (Fig. 7). However, topical application of both gentamicin and ZnO-NPs simultaneously increased the progression of wound healing in comparison with mice treated with gentamicin or ZnO-NPs alone. There was a noticeable improvement in wound closure of mice treated with a combination of gentamicin and ZnO-NPs at day 6 with complete wound healing at day 10 (Fig. 7 and Table 5). It is worth mentioning that no adverse effects were observed on mice body weight, general health or behavior during treatment. These results obviously indicate that ZnO-NPs have the potential to accelerate antibiotic action on skin wound promoting rapid closure and healing in wounds of *S. aureus*-infected mice.

**Table 5:** Effect of ZnO-NPs, gentamicin and their combination on wound area

**Mice group**	**Wound area (mm**^**2**^**)/ (% of wound contraction)**				
	**Days post treatment**				
	**Day zero**	**Day 3**	**Day 6**	**Day 10**	**Day 14**
**Control (Untreated mice)**	72 mm^2^	72 mm^2^	72 mm^2^	70 mm^2^	64 mm^2^
**ZnO-NPs treated mice**	72 mm^2^	48 mm^2^ (33.3%)	30 mm^2^ (58.3%)	15 mm^2^ (79.1%)	0 mm^2^ (100%)^a^
**Gentamicin treated mice**	72 mm^2^	45 mm^2^ (37.5%)	25 mm^2^ (65.3%)	10 mm^2^ (86.1%)	0 mm^2^ (100%)^a^
**ZnO-NPs + gentamicin treated mice**	72 mm^2^	21 mm^2^ (70.8%)	5 mm^2^ (93%)	0 mm^2^ (100%)^a^	0 mm^2^ (100%)^a^

^a^Complete healing

## Discussion

*S. aureus* infection has been alarming mainly due to its resistance to multiple antibiotics. Bacterial ability to form biofilm is considered a big challenge that enhances bacterial capacity to resist antimicrobial agents making bacterial eradication is very difficult [26]. In the current study, the ability of *S. aureus* isolates to form biofilm was quantitatively evaluated. Approximately 99% of *S. aureus* isolates were capable of forming biofilms with variable degrees. Noticeably, 54.8% of *S. aureus* isolates were found to be strong biofilm producers. These findings could account for the higher resistance rates developed by *S. aureus* isolates to various antibiotics tested herein.

Failure to develop new antibiotics, combined with the spread of microbial resistance, may lead to increased morbidity and mortality, especially in health care facilities. Novel strategies for prevention and eradication of bacterial biofilm are urgently required to modify the traditional treatments. In the present study, the approach of using nanoparticles in inhibition of bacterial biofilms was characterized comprehensively. One of the most important used nanoparticles as antibiofilm agent is ZnO-NPs. ZnO-NPs have been previously evaluated for their antibacterial and antibiofilm activity against *S. aureus* and *P. aeruginosa*. The antibiofilm and antibacterial activity of ZNO-NPs is thought to be due to the production of reactive oxygen species (ROS) such as superoxide anion, hydroxyl radicals, and hydrogen peroxide that negatively affect bacterial cells [27]. Accumulation of ZnO-NPs in the outer membrane of bacterial cell trigger Zn^2+^ release, which would cause bacterial cell membrane disintegration, membrane protein damage and genomic instability resulting in the death of bacterial cells. Furthermore, ZnO-NPs suppress amyloid peptide fibrillation essential for bacterial biofilm formation [28]. In addition to the antibacterial potential, it has been shown that ZnO-NPs could possess a potent antiviral activity. The antiviral activity of ZnO-NPs has been shown to be related to prevention of viral entry, viral replication and spreading to organs which can eventually trigger ROS leading to oxidative injury and viral death [29]. Furthermore, zinc containing compounds exhibit antiviral activity through attachment to virus, inhibition of virus infection and uncoating in addition to inhibition of viral polymerases and protease. Finally, the antiviral activity of ZnO-NPs against HSV-1 could be due to trapping the virions and hence block the viral entrance into target cells as well as the selective inhibitory effect of Zn^+2^ on viral polymerase [30].

The antibiofilm activity of ZnO-NPs against *S. aureus* ATCC 6538 and four bacterial isolates recovered from different clinical sources; burn, endotracheal aspirate, wound and ear infection, was fully characterized. Importantly, ZnO-NPs exhibited a good antibiofilm activity against *S. aureus*. This antibiofilm activity was found to be concentration-dependent, which further confirms its efficiency as antibiofilm agents. The antibiofilm activity of ZnO-NPs can be explained by interference with bacterial biofilm integrity. ZnO-NPs affect exopolysaccharide synthesis that plays a crucial role in bacterial initial adhesion to host cells and development of a complex biofilm. Moreover, it was shown that ZnO-NPs could easily penetrate bacterial cell wall and kill the harmful microorganism quickly [31].

Current results indicate a synergistic antibiofilm effect in all combinations between ZnO-NPs and tested antibiotics. This synergistic effect could be due to the interaction between antibiotics and ZnO-NPs. Nanoparticles have large surface area which allows them to closely interact with antibiotics. Antibiotic molecules that contain active groups like hydroxyl and amido groups can easily react with ZnO-NPs by chelation and consequently their diffusion and penetration through biofilm and exhibiting their antimicrobial activity against bacterial cells is largely improved. Ryan *et al*. (2001) reported that ZnO-NPs could inhibit efflux pump system that mediates bacterial resistance to different antibiotics [32]. In addition, ZnO-NPs induce production of free radicals that significantly attach to thiol-containing proteins in bacterial cell wall and ultimately resulting in higher degradation potency [33].

Targeting cell surface hydrophobicity is a novel way of inhibiting biofilm formation. Cell surface hydrophobicity and exopolysaccharide play an important role in bacterium-host cell interactions and biofilm architectures in microbes. Previous reports confirm that interference with cell surface hydrophobicity would help combat biofilm production in different microorganisms including *S. aureus*. Furthermore, the increased hydrophobicity of bacterial cell surface could result in improved cell adherence to various surfaces such as mucosal epithelial cells and phagocytes [34]. Current results show that treating *S. aureus* with ZnO-NPs reduces cell surface hydrophobicity as compared to untreated cells. These finding agreed with Pati *et al.* (2014) who reported that treatment with ZnO-NPs reduces cell surface hydrophobicity of both *S. aureus* and *P. aeruginosa* and leads to inhibition of biofilm formation [35].

The effect of ZnO-NPs on *S. aureus* virulence was investigated herein. *S. aureus* produces a plethora of extracellular virulence factors and enzymes to invade and establish infections in the host system. The α-hemolysin is one such kind of exotoxin which is highly potent in lysis of various host cells such as erythrocytes, macrophages, monocytes, endothelial cells and epithelial cells. Importantly, *S. aureus*-induced lysis of RBCs was greatly reduced upon treatment of bacterial cells with ZnO-NPs. It is possible that ZnO-NPs bind to bacteria and mask the ligands making them less accessible for binding to RBCs [35].

Biofilms represent the major challenging problem for treatment of catheter-associated urinary tract infections (CAUTIs). *S. aureus* is an opportunistic pathogen that is considered a significant uropathogen in CAUTIs [36]. Importantly, treatment with ZnO-NPs significantly reduced total number of *S. aureus* biofilm cells on catheter as compared to control untreated catheter. These findings indicate that ZnO-NPs inhibited biofilm formation and thus could be effective in preventing catheter-related urinary tract infections and have the potential to control biofilms in device-associated *S. aureus* infections.

The gene expression profiles of ZnO-NPs treated *S. aureus* were studied in order to further investigate the inhibitory mechanism of ZnO-NPs on bacterial biofilm formation. Interestingly, exposure of *S. aureus* to ZnO-NPs affects the expression of genes involved in both biofilm formation and oxidative stress response. The expression of biofilm-related genes (*icaA* and *sarA*) and oxidative stress gene *katA* as well as the general regulator *sigB* was significantly repressed in ZnO-NPs treated bacteria as compared to untreated cells. On the other hand, the expression of accessory gene regulator; *agrA* was significantly induced in *S. aureus* upon treatment with ZnO-NPs. It has been reported that *agr* is a dominant regulator of biofilm development and formation. Activation of *agr* induces dispersion *S. aureus* cells to transit to planktonic state, so biofilm initiation, adhesion and maturation require low *agr* transcription. Numerous studies on *S. aureus* have shown that *agrA* mutants possessed increased capacity to form biofilm [37, 38] which agreed with current observations that the inhibition of biofilm formation by ZnO-NPs could be due to induced expression of *agrA*.

The staphylococcal accessory regulator, *sarA* is a well-known global master regulator of biofilm and virulence genes in *S. aureus*. Present results revealed the downregulation of *sarA* in *S. aureus* upon ZnO-NPs treatment. It is well known that intracellular adhesion genes *icaA* and *icaD* are positively regulated by *sarA*. Both *icaA* and *icaD* are involved in slime synthesis and therefore play an important role in *S. aureus* biofilm formation [39]. Decreased expression of *icaA* and *icaD* in *S. aureus* following treatment with ZnO-NPs could account for the impairment of biofilm formation observed herein. In addition, expression of MSCRAMMs that are involved in the initial stage of biofilm formation including FnbA, FnbB and ClfA are regulated by *sarA* [40, 41], which further supports that reduced expression of *sarA* has a negative impact on bacterial biofilm formation by *S. aureus*.

Reduced biofilm formation in ZnO-NPs treated *S. aureus* could be further explained by repression of *sigB* in cells upon treatment with ZnO-NPs. *S. aureus* mutants in the global regulator *sigB* are unable to form a biofilm *in vitro* [42]. It is possible that *sigB* inhibition will also disperse an established biofilm. Repression of *sigB* in established *S. epidermidis* biofilms led to a pronounced dispersal event, suggesting that a similar mechanism may occur in *S. aureus*. Finally, treatment of *S. aureus* with ZnO-NPs resulted in the downregulation of catalase, which further account for the antibacterial efficiency of ZnO-NPs. *S. aureus* expresses a number of proteins that play a critical role in protecting cells against different stresses especially oxidative stress. For instance, *KatA* enables *S. aureus* to survive oxidative stress through inactivation of H_2_O_2_ and interrupting the formation of toxic ROS [43].

The influence of ZnO-NPs on *S. aureus* pathogenesis was evaluated herein using mice infection model. Treating *S. aureus* with ZnO-NPs markedly affects its pathogenesis in host. Current findings indicate that ZnO-NPs could be used as topical anti-infective agent especially in combination with commonly used antibiotics such as gentamicin. Mechanism of ZnO-NPs underlying this effect may be due to production of ROS that damage bacterial cells, interference with biofilm integrity by interrupting exopolysaccharide synthesis that plays a crucial role in development of a complex biofilm [44]. Moreover, interruption of bacterial energy transduction and enzyme activity as well as bacterial DNA synthesis by ZnO-NPs could lead to enhanced bacterial clearance during wound healing and make bacterial cells more susceptible to antibiotic treatment [45].

## Conclusion

In this work, the antibacterial and antibiofilm activity of ZnO-NPs against *S. aureus* have been extensively characterized both *in vitro* and *in vivo.* Interestingly, ZnO-NPs exhibited a potent antibiofilm activity against *S. aureus*. Furthermore, ZnO-NPs significantly enhanced *S. aureus* susceptibility toward various antibiotics and markedly reduced its pathogenesis in host. Importantly, the present study shed light on the antibacterial and antibiofilm mechanisms of ZnO-NPs. For instance, ZnO-NPs exhibit antibiofilm activity against *S. aureus* through affecting bacterial cell surface hydrophobicity and genes involved in biofilm formation. The antivirulent potential of ZnO-NPs observed herein could be attributed to the down regulation of *S.aureus* virulent genes upon exposure to ZnO-NPs. In addition, *S. aureus*-induced lysis of RBCs was greatly reduced following treatment of bacterial cells with ZnO-NPs. Finally, ZnO-NPs markedly impaired *S. aureus* pathogenesis in mice infection model. The findings of current study greatly extend our knowledge about ZnO-NPs as a potent antibiofilm and antivirulent agent. In summary, current study in addition to the previously published ones further support the promising role of ZnO-NPs as antibacterial and antibiofilm agent especially against the important pathogen *S. aureus.* ZnO-NPs could be considered as a valuable adjuvant in combination therapy with traditionally used antibiotics.

## Supplementary Information

**Additional file 1: Supplementary Table S1**. Comparison between different types of nanomaterials based on their advantages and disadvantages.

**Additional file 2. Supplementary Table S2.** Susceptibility profiles of *S. aureus* isolates against various antimicrobial agents.

**Additional file 3: Supplementary Table S3**. MIC values for both antibiotics and ZnO-NPs against *S. aureus.*

## Data Availability

The authors confirm that the data supporting the findings of this study are available within the article.

## Abbreviations

MH broth: Mueller Hinton
S: Susceptible
I: Intermediate
R: Resistant
MIC: Minimum inhibitory concentration
ZnO-NPs: Zinc oxide nanoparticles
mm: Millimeter
OD: Optical density
MBIC: Minimum biofilm inhibitory concentration
TS: Trypticase soy
FICI: Fractional inhibitory concentration index
HI: Hydrophobicity index
MDR: Multidrug resistant
RBCs: Red blood cells
ROS: Reactive oxygen species
