# Supplementary material for: Zinc oxide nanoparticles reduce biofilm formation, synergize antibiotics action and attenuate Staphylococcus aureus virulence in host; an important message to clinicians
Source: BMC Microbiol. 2022 Oct 11;22:244. doi: 10.1186/s12866-022-02658-z (PMC9552502; doi:10.1186/s12866-022-02658-z)
Supplement: Supplementary file 1 — Additional file 1: Supplementary Table S1. Comparison between different types of nanomaterials based on their advantages and disadvantages. [file 12866_2022_2658_MOESM1_ESM.docx]

**Supplementary Table S1:** Comparison between different types of nanomaterials based on their advantages and disadvantages

| **Type of nanomaterial** | **Advantage** | **Disadvantage** | **References** |
| --- | --- | --- | --- |
| **Lipid (liposome)** | **1-** Most used nanotechnology-based delivery systems for small molecules as peptides.  **2-** Used as carriers for delivering drugs.  **3-** High therapeutic index.  **4-** Have a protective effect on incorporated drugs.  **5-** Included PEGylated lipids to bypass the reticuloendothelial system (RES) & promote accumulation in tumors & to mitigate or suppress immune response.  **6**- Biocompatible. | **1-** Concentration of lipophilic drugs should be increased.  **2-** Toxicity for example:- cationic liposomes can interact with serum proteins, leading to systemic toxicity.  **3-** Impairment of RES function, hepatomegaly, granulomas & splenomegaly at higher doses  **4-** Trigger of immune system. | [1, 2] |
| **Carbon based nanomaterial (CNTs)** | **1-** Very dynamic and used for drug delivery system.  **2-** Their biological and chemical properties allow passive diffusion of CNTs across lipid bilayer, or attachment to cell.  **3-** Possess electrical and thermal conductivity.  **4-** Interesting media for electrical energy storage. | **1-** Limited use due to toxicity (could be source of occupational lung diseases in workers of CNT industries).  **2-** Non biodegradable | [1, 3] |
| **Polymeric based nanomaterial (natural polymeric material as starch and chitosan & synthetic polymeric material as polylactic and Poly β-hydroxybutyrate)** | **1-** Deliver agents at higher conc. & minimize toxicity.  **2-** Highly effective.  **3-** Protect drugs against degradation.  **4-** In medical applications & tissue engineering.  **6-** Able to cross blood-brain barrier (BBB).  **7-** Biocompatible, biodegradable & cost effective. | **1-** Natural polymeric materials possess poor water solubility, low porosity and poor cell attachment.  **2-** Synthetic polymers are Poor toughness, degradation speed is slow. In addition, high crystallinity and bad thermal stability.  **3-** Low yield of payload loading and burst release. | [4, 5] |
| **Ceramic nanoparticle**  **silica (SiO_2_)** | **1-** Silica NPs are appropriate carriers because of biocompatibility, ease of synthesis & surface modification.  **2-** Good candidates for drug carriers due to small size & surface modification.  **3-** Controlled release of drugs. | **1-** Toxicity, immunogenicity, route of administration, dissociation of polymers, and clearance, should be considered for safety. e.g. mesoporous silica NPs (MSNs) have high density of silanol groups on surface interact with phospholipids of RBCs leading to hemolysis. Surface modification is recommended by biocompatible polymers such as PEG.  **2-** Low stability & formation of aggregates. | [6, 7] |
| **Metallic or metal oxide NPs copper oxide (CuO), titanium dioxide (TiO_2_), iron oxide (Fe_2_O_3_), (MgO) & aluminum oxide (Al_2_O_3_)** | **1-** Increased surface area.  **2-** Small size allows longer circulation & tissue penetration.  **3-** Multiple applications (targeting, diagnostics, and therapy). | **1-** Limited biocompatibility & cytotoxicity.  **2-** Non biodegradability. | [1] |
| **Iron oxide NPs** | **1-** Ultrafine size & biocompatibility  **2-** Promising candidates for various biomedical applications. | **1-** Cytotoxicity | [8, 9] |
| **Silver (Ag) NPs** | **1-** Biocompatible & bioavailable.  **2-** Antibacterial activity.  **3-** Anticancer properties. | **1-** Combination of chitosan & polyvinyl pyrrolidone (PVP) polymers were coated by AgNPs to improve biocompatibility, biodegradability, antibacterial & wound healing.  **2-** Ag-NPs affect central nervous system & cause neuroinflammation by inducing release proinflammatory cytokines & produce ROS and nitric oxide.  **3-** Cytotoxic effects on osteoclasts, osteoblasts, cardiovascular & respiratory systems, DNA & embryo development abnormalities. | [10, 11] |
| **Gold (Au) NPs** | **1-** Biocompatibility & bioavailability.  **2-** Higher antibacterial activity.  **3-** Diagnosis of diseases & as vehicles for delivering other effective agents to specific locations.  **4-** Low toxicity. | **1-** Despite of the advantageous characteristics of AuNPs, this material is not biodegradable, therefore some concerns have questioned about the impact of gold accumulation inside the body & interaction with immune system.  **2-** High cost. | [12, 13] |
| **ZnO-NPs** | **1-** One of the most important metal oxide NPs & employed in various fields due to peculiar physical & chemical properties.  **2-** Because of strong UV absorption properties, they are increasingly used in personal care products, such as cosmetics and sunscreen.  **3-** ZnO-NPs have superior antibacterial, antimicrobial, and excellent UV blocking properties.  **4-** Compared with other metal oxide, ZnO-NPs is comparatively inexpensive, relatively less toxic property exhibit excellent biomedical applications, such as anticancer, drug delivery, antibacterial, diabetes treatment, anti-inflammation, wound healing & bioimaging.  **5-** Famous for catalytic efficiency, chemical stability & strong adsorption ability.  **6-** ZnO NPs have advantages over Ag NPs, as low production cost, white appearance & UV-blocking properties. | **1-** Cytotoxicity could be occurred at high concentration & large particle size. | [14-16] |

**References**

1. Lamberti, M., et al., Advantages and risks of nanotechnologies in cancer patients and occupationally exposed workers. Expert opinion on drug delivery, 2014. 11(7): p. 1087-1101.

2. Maja, L., K. Željko, and P. Mateja, Sustainable technologies for liposome preparation. The Journal of Supercritical Fluids, 2020. 165: p. 104984.

3. Johnston, H.J., et al., A critical review of the biological mechanisms underlying the in vivo and in vitro toxicity of carbon nanotubes: The contribution of physico-chemical characteristics. Nanotoxicology, 2010. 4(2): p. 207-246.

4. Kaushik, S., Polymeric and Ceramic Nanoparticles: Possible Role in Biomedical Applications, in Handbook of Polymer and Ceramic Nanotechnology. 2021, Springer. p. 1293-1308.

5. Alavi, M. and R.S. Varma, Antibacterial and wound healing activities of silver nanoparticles embedded in cellulose compared to other polysaccharides and protein polymers. Cellulose, 2021. 28(13): p. 8295-8311.

6. Alavi, M., S. Thomas, and M. Sreedharan, Modification of silica nanoparticles for antibacterial activities: mechanism of action. Micro Nano Bio Aspects, 2022. 1(1): p. 49-58.

7. Nguyen, T.L., Y. Choi, and J. Kim, Mesoporous silica as a versatile platform for cancer immunotherapy. Advanced Materials, 2019. 31(34): p. 1803953.

8. Zhao, X., et al., Ultrasmall superparamagnetic iron oxide nanoparticles for magnetic resonance imaging contrast agent. Journal of nanoscience and nanotechnology, 2014. 14(1): p. 210-220.

9. Soenen, S.J., et al., Investigating the toxic effects of iron oxide nanoparticles, in Methods in enzymology. 2012, Elsevier. p. 195-224.

10. Ozdal, M. and S. Gurkok, Recent advances in nanoparticles as antibacterial agent. ADMET and DMPK, 2022. 10(2): p. 115-129.

11. Alavi, M., et al., Various novel strategies for functionalization of gold and silver nanoparticles to hinder drug-resistant bacteria and cancer cells. Micro Nano Bio Aspects, 2022. 1(1): p. 38-48.

12. Vinhas, R., et al., Gold nanoparticle-based theranostics: disease diagnostics and treatment using a single nanomaterial. Nanobiosensors in Disease Diagnosis, 2015. 4: p. 11.

13. Dykman, L.A. and N.G. Khlebtsov, Immunological properties of gold nanoparticles. Chemical science, 2017. 8(3): p. 1719-1735.

14. Jiang, J., Pi, J., & Cai, J. (2018). The advancing of zinc oxide nanoparticles for biomedical applications. Bioinorganic chemistry and applications, 2018.

15. Elshama, S.S., M.E. Abdallah, and R.I. Abdel-Karim, Zinc oxide nanoparticles: therapeutic benefits and toxicological hazards. The Open Nanomedicine Journal, 2018. 5(1).

16. Melk, M.M., et al., Antiviral Activity of Zinc Oxide Nanoparticles Mediated by Plumbago indica L. Extract Against Herpes Simplex Virus Type 1 (HSV-1). International Journal of Nanomedicine, 2021. 16: p. 8221.
