# Supplementary material for: Zinc oxide nanoparticles reduce biofilm formation, synergize antibiotics action and attenuate Staphylococcus aureus virulence in host; an important message to clinicians
Source: BMC Microbiol. 2022 Oct 11;22:244. doi: 10.1186/s12866-022-02658-z (PMC9552502; doi:10.1186/s12866-022-02658-z)
Supplement: Supplementary file 2 — Additional file 2. Supplementary Table S2. Susceptibility profiles of S. aureus isolates against various antimicrobial agents. [file 12866_2022_2658_MOESM2_ESM.docx]

**Supplementary Table S2: Susceptibility profiles of *S. aureus* isolates against various antimicrobial agents**

| **Antibiotic/Susceptibility** | | | | | | | | | | | | | |
| --- | --- | --- | --- | --- | --- | --- | --- | --- | --- | --- | --- | --- | --- |
| **Isolate No** | **LZD^1^** | **SXT** | **DA** | **ME** | **CIP** | **TE** | **C** | **CTX** | **CRO** | **AZM** | **VA** | **CN** | **E** |
| ***S. aureus* ATCC 6538** | S | S | S | S | S | S | S | S | S | S | S | S | S |
| **1B^2^** | S | S | S | R | S | R | S | I | I | S | S | R | S |
| **2B** | S | S | S | R | S | S | S | S | S | S | S | S | S |
| **3B** | S | S | S | R | S | R | S | R | I | S | S | R | S |
| **4B** | S | S | S | R | S | R | S | R | R | S | S | R | S |
| **5B** | S | S | S | R | S | I | R | I | R | S | S | S | S |
| **6B** | S | S | S | R | S | S | S | R | R | S | S | S | S |
| **7B** | S | S | S | R | S | R | S | S | S | R | S | S | R |
| **8B** | S | S | S | R | S | R | S | I | I | S | S | R | S |
| **9B** | S | S | S | R | S | I | R | R | I | R | S | S | R |
| **10B** | S | S | s | R | S | R | R | R | R | R | S | S | R |
| **11B** | S | S | S | R | S | R | S | I | I | R | S | S | R |
| **12B** | S | S | S | R | S | R | R | R | R | R | S | S | R |
| **13B** | S | S | S | R | R | R | R | R | R | S | S | R | S |
| **14B** | S | S | S | R | S | R | S | I | I | S | S | R | S |
| **15B** | S | S | S | R | S | S | S | R | R | S | S | S | S |
| **16B** | S | S | S | R | S | S | S | R | R | S | S | S | S |
| **17B** | S | S | S | R | S | S | S | R | R | R | S | S | I |
| **18B** | S | S | S | R | S | S | S | R | R | S | S | S | S |
| **19B** | S | S | S | R | S | R | R | R | R | S | S | S | S |
| **20B** | S | S | S | R | S | R | R | R | R | S | S | S | S |
| **21B** | S | S | S | R | R | R | S | R | R | S | S | R | S |
| **22B** | S | S | S | R | S | R | S | I | I | S | S | R | S |
| **23B** | S | S | S | R | S | I | S | I | I | S | S | R | S |
| **24B** | S | S | S | R | R | R | R | R | R | R | S | R | R |
| **25B** | S | R | S | R | R | S | S | R | R | R | S | R | R |
| **26B** | S | S | R | R | R | R | R | R | R | R | S | R | R |
| **27B** | S | S | S | R | S | S | S | S | S | I | S | S | I |
| **28B** | S | S | S | R | S | R | S | I | I | S | S | R | S |
| **29B** | S | S | S | R | S | S | S | R | R | S | S | S | S |
| **30B** | S | S | S | R | S | R | S | R | R | R | S | S | R |
| **31B** | S | S | S | R | S | R | R | R | R | S | S | S | S |
| **32B** | S | R | S | R | R | R | S | R | R | R | S | R | R |
| **33B** | S | S | S | R | S | S | S | S | I | S | S | S | S |
| **34B** | S | S | S | R | S | I | S | I | I | S | S | R | S |
| **35B** | S | S | S | R | S | S | S | I | I | S | S | S | S |
| **36B** | S | S | S | R | S | S | S | R | R | S | S | S | S |
| **37B** | S | S | S | R | S | R | S | I | I | S | S | R | S |
| **38B** | S | S | R | R | R | R | R | R | R | R | S | R | R |
| **39B** | S | S | S | R | R | R | R | R | R | S | S | R | S |
| **40B** | S | S | S | R | S | R | S | R | R | I | S | S | I |
| **41B** | S | S | S | R | S | I | S | I | I | S | S | S | S |
| **42B** | S | S | S | R | S | I | S | I | I | S | S | R | S |
| **43B** | S | S | S | R | S | R | S | R | I | S | S | R | S |
| **44B** | S | S | S | R | S | S | S | I | I | S | S | S | S |
| **45B** | S | S | S | R | S | R | S | R | R | S | S | R | S |
| **46U** | S | S | S | R | S | S | S | S | S | R | S | S | R |
| **47U** | S | S | S | R | S | R | R | R | R | S | S | S | S |
| **48U** | S | S | S | R | S | S | S | R | R | S | S | S | S |
| **49U** | S | S | S | R | S | S | S | I | I | S | S | S | S |
| **50U** | S | S | S | R | S | R | S | R | R | S | S | S | S |
| **51U** | S | S | S | R | S | R | R | I | I | S | S | R | S |
| **52U** | R | S | R | R | S | S | S | I | I | I | S | R | R |
| **53EY** | S | S | S | R | S | S | S | R | R | S | S | S | S |
| **54EY** | S | S | S | R | S | I | S | I | I | S | S | R | S |
| **55EY** | S | S | S | R | S | R | S | I | I | S | S | R | S |
| **56EY** | S | S | S | R | S | S | S | R | R | S | S | S | S |
| **57EY** | S | S | S | R | S | S | S | I | I | S | S | S | S |
| **58EN** | S | S | S | R | S | I | S | R | R | S | S | S | S |
| **59EN** | S | S | S | R | R | R | S | R | R | S | S | R | S |
| **60EN** | S | S | S | R | S | I | S | I | I | S | S | R | S |
| **61EN** | S | S | S | R | R | S | S | I | I | R | S | S | R |
| **62EN** | S | S | S | R | R | S | S | I | I | R | S | S | R |
| **63EN** | S | S | S | R | R | S | S | I | I | R | S | S | R |
| **64EN** | S | S | S | R | S | S | S | I | S | S | S | S | S |
| **65EN** | S | S | S | R | S | R | S | I | I | S | S | R | S |
| **66EN** | S | S | S | R | R | R | S | R | I | S | S | R | S |
| **67EN** | S | S | S | R | S | S | S | R | R | S | S | S | S |
| **68EA** | S | S | S | R | S | S | S | R | I | I | S | S | R |
| **69EA** | S | S | S | R | S | R | S | R | R | R | S | R | R |
| **70EA** | S | R | S | R | R | R | S | R | R | R | S | R | R |
| **71W** | S | S | S | R | S | S | S | R | I | S | S | R | S |
| **72W** | S | S | S | R | S | R | S | I | I | S | S | R | R |
| **73W** | S | S | S | R | S | R | S | R | R | S | S | R | S |
| **74W** | S | S | S | R | S | R | S | I | I | S | S | R | S |
| **75W** | S | S | S | R | S | S | S | S | S | S | S | S | S |
| **76W** | S | S | S | R | S | R | S | S | S | R | S | S | R |
| **77W** | S | S | S | R | S | I | S | R | R | S | S | S | S |
| **78W** | S | S | S | R | S | R | S | I | I | S | S | R | S |
| **79W** | S | S | S | R | S | R | S | R | R | R | S | R | R |
| **80W** | S | S | S | R | S | S | S | I | I | S | S | R | S |
| **81W** | S | S | S | R | R | R | S | I | I | S | S | R | S |
| **82W** | S | S | S | R | S | S | S | R | I | S | S | S | S |
| **83W** | S | S | S | R | S | I | S | R | I | S | S | S | S |
| **84W** | S | R | S | R | R | I | S | R | R | R | S | R | R |
| **85W** | S | S | S | R | S | R | R | I | I | S | S | S | S |
| **86W** | S | S | S | R | R | R | R | R | R | S | S | R | S |
| **87W** | S | S | S | R | S | S | S | R | I | S | S | R | S |
| **88W** | S | S | S | R | S | R | S | R | R | S | S | R | S |
| **89W** | S | S | S | R | S | R | R | R | R | S | S | S | S |
| **90W** | S | S | S | R | S | R | R | R | R | S | S | R | S |
| **91W** | S | S | S | R | S | I | S | R | R | R | S | S | R |
| **92W** | S | S | S | R | S | I | S | R | R | S | S | S | S |
| **93W** | S | S | S | R | S | S | S | R | R | S | S | S | S |
| **94W** | S | R | R | R | R | R | I | I | I | R | S | S | R |
| **95W** | S | S | S | R | S | S | S | R | R | S | S | S | S |
| **96W** | S | S | S | R | S | S | S | I | I | S | S | R | S |
| **97W** | S | S | R | R | R | R | R | R | R | R | S | R | R |
| **98W** | S | S | S | R | S | R | R | R | R | S | S | S | S |
| **99W** | R | R | R | R | R | S | R | R | R | R | S | R | R |
| **100W** | S | S | S | R | S | R | S | R | I | I | S | S | I |
| **101W** | S | S | R | R | R | R | I | R | R | R | S | R | R |
| **102W** | S | S | S | R | S | S | S | I | I | S | S | R | S |
| **103W** | S | S | S | R | S | R | S | I | I | S | S | R | S |

^1^ Linezolid (LZD, 3µg); sulfamethoxazole-trimethoprim (SXT, 25µg); clindamycin (DA, 2µg); methicillin (ME, 5µg); ciprofloxacin (CIP, 5µg); tetracycline (TE, 10µg); chloramphenicol (C, 30µg); cefotaxime (CTX, 30µg); ceftriaxone (CRO, 30µg); azithromycin (AZM, 15µg); vancomycin (VA, 30µg); gentamicin (CN, 10µg); erythromycin (E, 15µg).

^2^ **B**; burn, **U**; urine, **EY**; eye, **EN**; endotracheal aspirate, **EA**; ear, **W**; wound.
