# Supplementary material for: Zinc oxide nanoparticles reduce biofilm formation, synergize antibiotics action and attenuate Staphylococcus aureus virulence in host; an important message to clinicians
Source: BMC Microbiol. 2022 Oct 11;22:244. doi: 10.1186/s12866-022-02658-z (PMC9552502; doi:10.1186/s12866-022-02658-z)
Supplement: Supplementary file 3 — Additional file 3: Supplementary Table S3. MIC values for both antibiotics and ZnO-NPs against S. aureus. [file 12866_2022_2658_MOESM3_ESM.docx]

**Supplementary Table S3: MIC values for both antibiotics and ZnO-NPs against *S. aureus***

| **Isolate No** | **Antibiotic/ MIC; µg/mL (Susceptibility)** | | | | | | |
| --- | --- | --- | --- | --- | --- | --- | --- |
|  | **Gentamicin S I R**  **≤ 4 8 ≥16** | **Ciprofloxacin**  **S I R**  **≤ 1 2 ≥4** | **Clindamycin**  **S I R**  **≤ 0.5 1-2 ≥4** | **Chloramphenicol**  **S I R**  **≤ 8 16 ≥32** | **Cefotaxime**  **S I R**  **≤ 8 16-32 ≥32** | **Azithromycin**  **S I R**  **≤ 2 4 ≥8** | **ZnO-NPs** |
| ***S. aureus* ATCC 6538** | 4(S) | 2(I) | 0.03(S) | 4(S) | 128(R) | 0.5(S) | 128 |
| **1B** | 128(R) | 2(I) | 0.06(S) | 16(I) | 16(I) | 4(I) | 256 |
| **2B** | 32(R) | 0.125(S) | < .003(S) | 4(S) | 0.125(S) | 0.25(S) | 256 |
| **24B** | 1024(R) | 128(R) | 512(R) | 128(R) | 2048(R) | >1024(R) | 128 |
| **38B** | 1024(R) | 64(R) | 8(R) | 64(R) | 1024(R) | >1024(R) | 128 |
| **41B** | 2(S) | 1(S) | < .003(S) | 4(S) | 0.125(S) | 1(S) | 512 |
| **42B** | 64(R) | 0.25(S) | 0.25(S) | 0.5(S) | 0.125(S) | 1(S) | 128 |
| **44B** | 8(I) | 1(S) | 0.25(S) | 4(S) | 64(R) | 1(S) | 256 |
| **48U** | 4(S) | 8(R) | 0.03(S) | 4(S) | 256(R) | 64(R) | 32 |
| **55EY** | 64(R) | 0.25(S) | 0.015(S) | 4(S) | 0.125(S) | 0.5(S) | 512 |
| **62EN** | 8(I) | 16(R) | 0.03(S) | 8(S) | 32(I) | 32(R) | 64 |
| **63EN** | 8(I) | 16(R) | 0.5(S) | 32(R) | 128(R) | 32(R) | 256 |
| **68EA** | 0.5(S) | 32(R) | <.003(S) | 8(S) | 0.5(S) | 0.5(S) | 128 |
| **82W** | 2(S) | 0.5(S) | 0.015(S) | 8(S) | 16(I) | 0.5(S) | 512 |
| **83W** | 1(S) | 0.25(S) | 0.06(S) | 8(S) | 0.5(S) | 2(S) | 512 |
| **84W** | 32(R) | 16(R) | 0.03(S) | 4(S) | 1024(R) | 32(R) | 128 |
| **91W** | 8(I) | 1024(R) | 256(R) | 16(I) | 1024(R) | 512(R) | 128 |
| **97W** | 1024(R) | 32(R) | 256(R) | 64(R) | 1024(R) | >1024(R) | 128 |
| **99W** | 256(R) | 64(R) | 4(R) | 128(R) | 1024(R) | 32(R) | 128 |
| **101W** | 1024(R) | 64(R) | 1024(R) | 64(R) | 1024(R) | >1024(R) | 128 |
